# Phantom with Pulsatile Arteries to Investigate the Influence of Blood Vessel Depth on Pulse Oximeter Signal Strength

**DOI:** 10.3390/s120100895

**Published:** 2012-01-16

**Authors:** Norbert Stuban, Masatsugu Niwayama, Hunor Santha

**Affiliations:** 1 Department of Electronic and Electrical Engineering, Niwayama Laboratory, Shizuoka University, 3-5-1 Johoku Nakaku, Hamamatsu 432-8561, Japan; E-Mail: tmniway@ipc.shizuoka.ac.jp; 2 Department of Electronics Technology, Budapest University of Technology and Economics, Goldmann Gy. t. 3., Budapest H-1111, Hungary; E-Mail: santha@ett.bme.hu

**Keywords:** reflectance pulse oximetry, oximeter, phantom, plethysmograph, pulsatile, CW NIRS

## Abstract

This paper describes a three-layer head phantom with artificial pulsating arteries at five different depths (1.2 mm, 3.7 mm, 6.8 mm, 9.6 mm and 11.8 mm). The structure enables formation of spatially and temporally varying tissue properties similar to those of living tissues. In our experiment, pressure pulses were generated in the arteries by an electronically controlled pump. The physical and optical parameters of the layers and the liquid in the artificial arteries were similar to those of real tissues and blood. The amplitude of the pulsating component of the light returning from the phantom tissues was measured at each artery depth mentioned above. The build-up of the in-house-developed pulse oximeter used for performing the measurements and the physical layout of the measuring head are described. The radiant flux generated by the LED on the measuring head was measured to be 1.8 mW at 910 nm. The backscattered radiant flux was measured, and found to be 0.46 nW (0.26 ppm), 0.55 nW (0.31 ppm), and 0.18 nW (0.10 ppm) for the 1.2 mm, 3.7 mm and 6.8 mm arteries, respectively. In the case of the 9.6 mm and 11.8 mm arteries, useful measurement data were not obtained owing to weak signals. We simulated the phantom with the arteries at the above-mentioned five depths and at two additional ones (2.5 mm and 5.3 mm in depth) using the Monte Carlo method. The measurement results were verified by the simulation results. We concluded that in case of 11 mm source-detector separation the arteries at a depth of about 2.5 mm generate the strongest pulse oximeter signal level in a tissue system comprising three layers of thicknesses: 1.5 mm (skin), 5.0 mm (skull), and >50 mm (brain).

## 1. Introduction

Oximeters measure the intensity of two or more light beams which are modulated by arterial pulsation. Light beams have different wavelengths, usually in the region of 650–950 nm. If the good arterial perfusion area is close to the skin surface, the signal strength of a reflective-type pulse oximeter is greater. However, in some cases, observation of deeper areas may be necessary. Studies show that it may be possible to determine the oxygen saturation of a fetus even through the abdomen of the mother [1]. Several papers on multilayer tissue phantoms support the field of pulse oximetry and have led to a better understanding of light travel through living tissues [2]. The source-detector distance, influence of the melanin content [3], simulated path of photons through the skin [4] and other areas were investigated with phantoms and theoretical analysis. Although the structure and parameters of the model for the simulation can easily be modified, analysis with smaller structural changes (<0.05 mm) is more difficult because the calculation requires a large volume of three dimensional model elements and a significant runtime. Therefore, a dynamic phantom could be suitable for examining temporal signal variations of a pulse oximeter, especially with weak pulsating arteries or thin arteries. In previous papers [2–4] multilayer phantoms with physical and optical parameters similar to those of real tissue were built or simulated. However, our study represents the first to use a realistic phantom with cylindrical pulsatile arteries at different depths. Because the pulsation of arteries is of fundamental importance in pulse oximetry, the authors worked out a method to build a dynamic phantom in which the arteries are represented by resilient silicone tubes and the diameter of the arteries changes with time (throbs) just like real tissues [5].

In this paper, a novel three-layer head phantom (skin, skull, and brain) with artificial arteries at five different depths is described. The optical properties of the layers (reduced scattering coefficient μ_s_^′^, absorption coefficient μ_a_, and layer thickness) are based on values identified and selected from the literature. The influence of artery depth on the signal strength of a pulse oximeter was investigated using this phantom. Pulse oximeters calculate the arterial oxygen saturation (SpO_2_ level) from the amplitudes of the measured red and infrared photo-plethysmographic waveforms [6].

### 1.1. Definition

Radiant flux or radiant power is the measure of the total power of electromagnetic radiation (e.g., light). It may be the total power emitting from a source or incident on a particular surface. The SI unit of radiant flux is W [7]. Although its exact definition is given by the SI, even large manufacturers often use misleading units for radiant flux in the datasheets of their optoelectronic products. Therefore, to avoid confusion, we have clarified the definition and unit.

## 2. Methods

The total radiant flux of the LED on the measuring head of an in-house-developed pulse oximeter was determined. To enable calculation of the absolute value of the radiant flux from the measured photodiode current, the sensitivity of the photodiode on the measuring head was determined. The absolute value of the pulsating component of the backscattered light at different artery depths was obtained. In addition, the rate of the incident and backscattered radiant flux was expressed by normalizing the absolute values with the total radiant flux of the LED. Thus, the numerical results became independent of the optical power of the LED. The in-house-developed pulse oximeter was fixed on a head phantom and the backscattered radiant flux was measured at different artery depths. All measurements were performed with a 910 nm wavelength LED (Roithner SMT660/910) as light source.

### 2.1. Phantom

**Figure 1.**
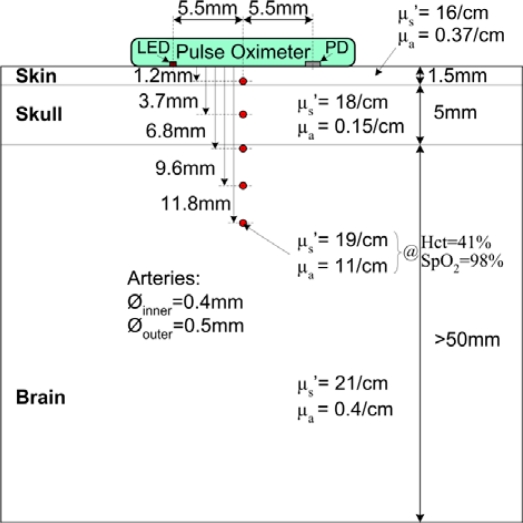
Structure of the head phantom constructed for the measurements. It comprises three tissue layers (skin, skull, and brain) and five arteries at different depths. The optical properties (absorption coefficient and reduced scattering coefficient) and physical properties (layer depth, artery diameter) are indicated in the figure.

The constructed head phantom consists of three tissue layers (skin, skull, and brain) and five arteries at different depths (1.2 mm, 3.7 mm, 6.8 mm, 9.6 mm and 11.8 mm). At each depth there is one artery running along the phantom. Figure 1 shows the layer structure and optical properties.

Several research groups have measured the optical properties of various human tissues using different equipment and slightly different methods. Different research groups came up with significantly different sets of results [8,9]. Because the methodological differences and the skin- and tissue-type variance among real subjects can lead to varied results, it is essential to carefully choose the optical properties for the simulation and experimental setup. We considered the methods and results of different research groups, and used Bashkatov’s results for the skull and skin properties. Bashkatov determined the absorption coefficient and reduced scattering coefficient of biological samples with an integrating sphere based method. They measured several tissue types and compared the result with those of other studies [10,11]. The absorption coefficient of the skull and skin at 910 nm are 0.15 cm^−1^ and 0.37 cm^−1^, respectively. Furthermore, the reduced scattering coefficients of the skull and skin at the same wavelength are 18 cm^−1^ and 16 cm^−1^, respectively. For the brain layer of the phantom, the optical properties of the gray matter measured by van der Zee *et al*. (μ_a_ = 0.4 cm^−1^ and μ_s_′ = 21 cm^−1^ at 910 nm) were selected [12]. The layers were prepared by mixing intralipid solution, purified water, gelatin and ink (Pilot INK-350-B). First the absorption coefficient was set up by adding ink to purified water while the absorption coefficient of the solution was continuously measured by a spectrophotometer. Then, the solution was heated up; gelatin was added, and boiled for two minutes. During cooling down, when the temperature of the mixture dropped to 42 °C, an appropriate amount of intralipid was added to adjust the scattering coefficient [13]. When the temperature dropped to 35 °C, the mixture was poured into a cube–shaped hard plastic receptacle (Figure 1) containing the prepared arteries. For each layer the procedure was repeated.

The arteries in the phantom were made of resilient silicone tubes (Aram 1175-04). The inner diameter of the arteries was 0.4 mm and the outer diameter was 0.5 mm (Figure 1). To ensure stable measurement conditions and reproducibility of the measurements, a solution of purified water and black ink (Pilot INK-350-B) was used instead of real blood. The hematocrit (Hct) value strongly affects the absorption and scattering of blood. In addition, the absorption changes with the SpO_2_ value. The scattering can be considered to be independent of the SpO_2_ value [14]. Based on the results of [14] we rescaled the absorption and scattering coefficients of blood by considering a hematocrit value of 41% and an SpO_2_ level of 98%. These values are accepted as normal values for a healthy adult subject. According to the rescaling, the absorption coefficient of the circulating blood in a living tissue is 11 cm^−1^ and the reduced scattering coefficient is 19 cm^−1^ at 910 nm (SpO_2_ = 98%, Hct = 41%). Using a spectrophotometer, the absorption coefficient of the ink-water solution was adjusted to this value. Next, the reduced scattering coefficient was adjusted by adding intralipid to the solution. As a result, the optical properties of the circulating liquid became identical to that of real blood at 910 nm. The absorption and scattering effects of the silicone tubes (artificial artery wall) were neglected. The relative position of the measuring head and the artery has a significant influence on the measured values. Thus, during the measurements the measuring head was always adjusted to the position indicated in Figure 1.

### 2.2. Pump

**Figure 2.**
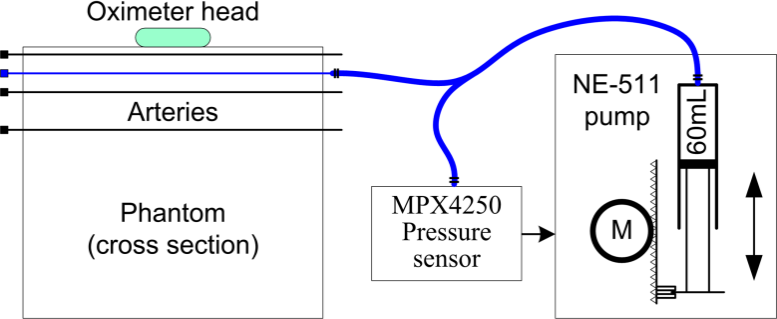
Generation of intra-arterial pressure waves in the phantom (emulating heart beats) by an electronically controlled pump. The pump can be connected to any of the arteries in the phantom with a Luer Lock fitting. The actual artery is marked in blue.

**Figure 3.**
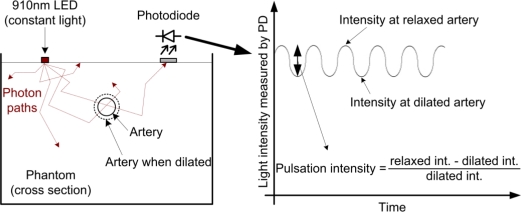
Photodiode signal sensing in an oscillating (*i.e.*, dilating and contracting) artery. An artery in the phantom is dilating and contracting due to the pulse waves generated by the pump. The volume change in the artery causes intensity change in the light passing through. This slight oscillation can be observed in the measured photodiode current (right side of the figure). Pulsation intensity refers to the strength of the oscillation.

An electronically controlled pump was built by mounting a precise pressure sensor (Freescale MPX4250) on a microcontroller-based syringe pump (type NE-511 from New Era Pumpsystem Inc.), as shown in Figure 2. Only one artery of the phantom was connected to the pump at any one time. With the continuous measurement of the arterial pressure the pump could produce a pressure waveform similar to that of an actual heartbeat. The frequency of the beats was 50–70/min during the measurements. The relationship between the applied pressure and inner diameter of the silicone tube was determined by our previously published method [5]. Based on this relationship, the dilation of the artery was controlled at every “heartbeat”. Pulsation intensity arose from the diameter change of the artery. When the pump increased the pressure in the artery, the diameter of the artery would increase commensurately, thus causing more liquid (blood) to flow. At 910 nm, the circulating liquid (blood) was a strong absorber compared with the other tissue types (see the absorption values in Figure 1). The greater the liquid amount in the optical path, the greater the amount of absorbed light, and thus, the smaller the light intensity measured by the photodiode. However, note that the actual pulsation and resultant oscillation of the light intensity were very small. This process is illustrated in Figure 3. On the left side, the cross section of an artery of the phantom in both relaxed and dilated states is shown. The light intensities at the relaxed and dilated states and the derived definition of the pulsation intensity are shown on the right side of the figure. The principle is the same as that of oximetry measurements on humans. All measurements described here were performed with a pulsation intensity of 4% [15], which means that the artery diameter measured in the dilated state was 4% greater than that measured in the relaxed state (0.4 mm).

### 2.3. The Pulse Oximeter Used for the Measurements

**Figure 4.**
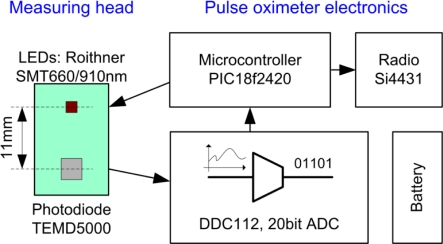
The structure of the in-house-developed direct converting pulse oximeter and its reflective-type measuring head (green). The distance between the photodiode and the light source (LED) on the measuring head was 11 mm. Note, that there were no intermediate electronic components between the photodiode and the analog-digital converter. The sensed current was directly digitized; consequently an outstanding noise performance was reached (SNR = 106 dB). To eliminate external noise sources the oximeter was battery powered, the measured data was transmitted in a wireless way, and the unit was encapsulated in an aluminum box.

The authors developed a direct current converting pulse oximeter. There were no analog components between the photodiode and the analog to digital converter (ADC). The current of the photodiode was directly digitized without any analog signal conditioning. This solution was feasible with the DDC112 ADC. Because of the lack of analog signal modification, the absolute value of the photodiode current could be measured. In addition, using the measured current, active area, and sensitivity of the photodiode, the incident radiant flux was calculated. The schematic structure of the pulse oximeter and the physical properties of the reflective-type measuring head can be seen in Figure 4. The LED was operated with an 8.1 mA continuous forward current, which resulted in 1.8 mW of radiant flux. The radiation half angle of the LED was ±55°. The LED chip with 660 nm wavelength was not used during measurements. The direct converting pulse oximeter transmitted the measured radiant flux values to a PC via a radio link (Si4431, SiLabs). The data evaluation software was coded in Matlab. According to our noise measurements, the signal to noise ratio of the in-house-developed direct converting pulse oximeter was 106 dB.

### 2.4. Determining the Radiant Flux

A TEMD5000 photodiode with an active area of 7.5 mm^2^ was mounted on the measuring head. For calculating the absolute value of the radiant flux from the measured photodiode current, the sensitivity of the photodiode at 910 nm was determined by a measurement series. A Hikoki 3664 optical power meter with a 9742 probe was used. The radiant flux reaching the photodiode was calculated from the ratio of the active area of the optical probe (92.16 mm^2^) and that of the photodiode (7.5 mm^2^). In addition, the total radiant flux of the LED of the measuring head was determined.

### 2.5. Simulation

The Lambert-Beer law is usually used for illustrating the principles of pulse oximetry [16]. Lambert-Beer law describes the propagation of light through a non-scattering medium; however, it does not describe the impacts of scattering. Because most tissues are highly scattering, we verified our experimental results using a three-dimensional Monte Carlo simulation [17] instead of calculations based on the Lambert-Beer’s law. In the simulation the anisotropy factor was g = 0.95, and the voxel size was 0.05 mm. The parameters of the simulation model and the properties of the phantom were identical and comprised three static layers with appropriate optical properties, together with seven arteries. In addition to the five arteries from the phantom model two additional arteries were employed to increase the spatial resolution in terms of depth. The distance between the light source and detector (11 mm) was the same as that of the pulse oximeter used for the phantom measurements. At each depth, the artery diameter was varied between 0.3 mm and 0.5 mm in increments of 0.05 mm. Linear curve fitting allowed the estimation of any diameters between 0.3 mm and 0.5 mm therefore, the simulation of a pulsatile signal with a defined amplitude was feasible. For each step, 10^7^ photons were vertically injected into the phantom at the source point. The size of the detector was assumed to be 7.5 mm^2^, which corresponded to the active area of the photodiode of the employed oximeter. The number of backscattered photons was recorded. By adjusting the pulsation intensity to 4%, the change in light intensity caused by the pulsation was calculated.

## 3. Results

### 3.1. Sensitivity of the Photodiode

**Figure 5.**
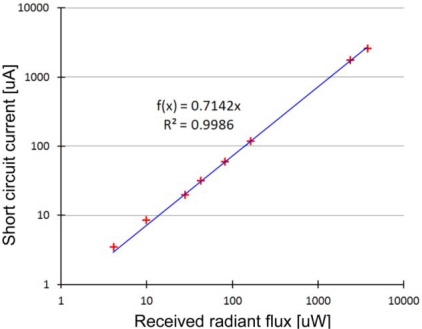
Sensitivity of the TEMD5000 photodiode with a 7.5 mm^2^ active area, measured on 910 nm. The measured data points at eight different levels of light are marked with red crosses. Linear regression (blue line) has a good fit.

The radiant flux received by the photodiode was measured at eight different levels of light intensity, while the short circuit current of the photodiode was also monitored. The results can be seen in Figure 5. Linear regression was applied to the eight data points with a good fit (R^2^ = 0.9986). According to our measurements, the sensitivity of the TEMD5000 photodiode is 0.71 A/W at 910 nm. The datasheet of the photodiode reports sensitivity at 950 nm only, which is 0.8 A/W.

Based on practical pilot measurements, the full-scale input of the ADC was set as 5,700 nA. This value prevents ADC saturation even in the case of strong environmental backlight. According to a noise measurement series, the real (noiseless) resolution of our pulse oximeter is 17 bits. The corresponding least significant bit (LSB) value is 44 pA. Based on the sensitivity of the photodiode, 44 pA is equivalent to 61.25 pW, which means that the pulse oximeter can measure radiant flux received by its photodiode with a resolution of 61.25 pW (LSB = 61.25 pW).

### 3.2. Phantom Measurements and Simulation

The pulsation intensity of the backscattered light was measured at five artery depths (Table 1). At the fifth artery depth (11.8 mm), the pulsation was indistinguishable from noise, and the measurement results for the 9.6 mm artery were also considered unreliable. To obtain useful numerical results, we expressed the measured data in terms of the radiant flux detected by the photodiode (nW, second column in Table 1). As mentioned above, the total radiant flux produced by the 910 nm LED was 1.8 mW. The detected radiant flux values were normalized to 1.8 mW and expressed in parts per million (ppm), as shown in the third column of the table; for example, at 6.8 mm artery depth, the pulsatile component of the sensed light is 0.1 ppm of the total radiant flux irradiated into the phantom by the LED.

The simulation results were normalized with the total number of injected photons (10^7^) and were also expressed in ppm. The simulation results are presented in the fourth column of the table with the difference between the measurement results and simulation results shown in the fifth column. At an artery depth of 11.8 mm, the path length of the photons in the artery was not adequate for obtaining a reliable statistical result.

**Figure 6.**
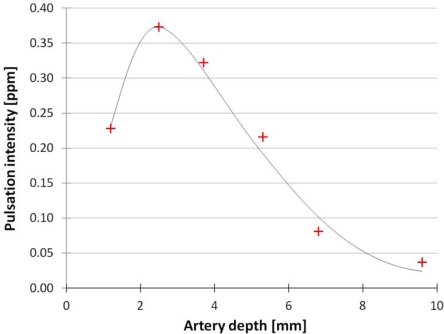
Simulated pulsation intensity in function of the artery depth. Successful simulation was carried out at six different artery depths, marked by red crosses. Around 2.5 mm depth the sensed pulsation intensity has a maximum point.

Figure 6 presents the simulation results. The strongest signal originates from the artery at 2.5 mm depth. The artery depth *vs.* the detected pulsation intensity shows a non-monotonic function, which means that the strongest signal does not originate from the most superficial artery. This phenomenon is caused by the optical path of the photons traveling in the tissues from the LED to the photodiode. Reuss [4] provides a graphical illustration of the photon path based on simulations. In [4], at a source-detector distance of 12 mm, the cross-section through which most photons travel appears to be at about 2.5 mm depth, which is a finding similar to our results. One must note, however, that the tissue structure used by [4] was different from the structure described in this paper.

## 4. Conclusions

A light beam passing through living tissues is modulated by arterial pulsation. This phenomenon is the basis of modern pulse oximeters. Using a phantom, we measured the amplitude of the pulsating component of the light returning from tissues at different artery depths. In addition, we performed Monte Carlo simulations under identical conditions. It was found that for a source-detector distance of 11 mm, the arteries at a depth of around 2.5 mm can be observed with the highest pulse oximeter signal level in a tissue system consisting of three layers of thicknesses 1.5 mm (skin), 5.0 mm (skull) and >50 mm (brain). The simulation results agree well with the measurement results with an error below 24% in each case. The differences probably originate from the practical uncertainties of the construction technology of the phantom and from the fact that the photon source in the simulation and the real LED have different intensity distributions that affect the optical paths of the photons. The limited number of photons used in our simulations could also contribute to the differences.

Determination of the sensitivity value of the TEMD5000 photodiode (0.71 A/W at 910 nm) can be useful for experts in the field because there are several types of Si PIN photodiodes with the same active area size (e.g., Vishay BPW34). Using the obtained sensitivity, the accuracy of the described phantom measurements has also been improved.

Accurate measurements of the detected light intensities are required to reliably determine arterial oxygen levels. The findings of this paper will lead to a better understanding of light propagation in biological tissues. Furthermore, the numerical results are useful for the design of pulse oximetry measurements and for estimating the required sensitivity of pulse oximeters currently under development.

## Figures and Tables

**Table 1.** Results of measurements (n > 20 in every case) performed on the phantom are shown in the second and third column. The fourth column shows the results of the simulation.

	**Pulsation Amplitude Resulted by**			
**Artery Depth [mm]**	**Phantom [nW]**	**Measurement [ppm]**	**Simulation [ppm]**	**Error**
1.2	0.46	0.26	0.23	14%
2.5	*	*	0.37	-
3.7	0.55	0.31	0.32	3.7%
5.3	*	*	0.22	-
6.8	0.18	0.10	0.08	23%
9.6	result may be invalid		0.04	-
11.8	no measurable data		invalid**	-

* no measurements were performed at this depth (only simulations);

** see the reason in the text.
